# Genome-scale metabolic model of *Rhodococcus jostii* RHA1 (*i*MT1174) to study the accumulation of storage compounds during nitrogen-limited condition

**DOI:** 10.1186/s12918-015-0190-y

**Published:** 2015-08-07

**Authors:** Mohammad Tajparast, Dominic Frigon

**Affiliations:** Microbial Community Engineering Laboratory, Department of Civil Engineering and Applied Mechanics, McGill University, 817 Sherbrooke Street West, Montreal, QC H3A 0C3 Canada

## Abstract

**Background:**

*Rhodococcus jostii* RHA1 growing on different substrates is capable of accumulating simultaneously three types of carbon storage compounds: glycogen, polyhydroxyalkanoates (PHA), and triacylglycerols (TAG). Under nitrogen-limited (N-limited) condition, the level of storage increases as is commonly observed for other bacteria. The proportion of each storage compound changes with substrate, but it remains unclear what modelling approach should be adopted to predict the relative composition of the mixture of the storage compounds. We analyzed the growth of *R. jostii* RHA1 under N-limited conditions using a genome-scale metabolic modelling approach to determine which global metabolic objective function could be used for the prediction.

**Results:**

The *R. jostii* RHA1 model (*i*MT1174) produced during this study contains 1,243 balanced metabolites, 1,935 unique reactions, and 1,174 open reading frames (ORFs).

Seven objective functions used with flux balance analysis (FBA) were compared for their capacity to predict the mixture of storage compounds accumulated after the sudden onset of N-limitation. Predictive abilities were determined using a Bayesian approach. Experimental data on storage accumulation mixture (glycogen, polyhydroxyalkanoates, and triacylglycerols) were obtained for batch cultures grown on glucose or acetate. The best FBA simulation results were obtained using a novel objective function for the N-limited condition which combined the maximization of the storage fluxes and the minimization of metabolic adjustments (MOMA) with the preceding non-limited conditions (max storage + environmental MOMA). The FBA solutions for the non-limited growth conditions were simply constrained by the objective function of growth rate maximization. Measurement of central metabolic fluxes by ^13^C-labelling experiments of amino acids further supported the application of the environmental MOMA principle in the context of changing environment. Finally, it was found that the quantitative predictions of the storage mixture during N-limited storage accumulation were fairly sensitive to the biomass composition, as expected.

**Conclusions:**

The genome-scale metabolic model analysis of *R. jostii* RHA1 cultures suggested that the intracellular reaction flux profile immediately after the onset of N-limited condition are impacted by the values of the same fluxes during the period of non-limited growth. PHA turned out to be the main storage pool of the mixture in *R. jostii* RHA1.

**Electronic supplementary material:**

The online version of this article (doi:10.1186/s12918-015-0190-y) contains supplementary material, which is available to authorized users.

## Background

Rhodococci are widely spread in many habitats such as soil, fresh water, seawater, and activated sludge wastewater treatment systems [1–4]. Their versatile metabolic activities make them good candidates for environmental bioremediation, and for a number of industrial applications including bio-desulfurization of fossil fuels [5–7], production of bio-surfactants [4], and production of acrylic acid [2], just to name a few. Specifically, *Rhodococcus jostii* RHA1 (RHA1) was isolated from lindane-contaminated soil and is best known for its superior ability to biodegrade polychlorinated biphenyls (PCBs) [8]. The genome of RHA1 has been completely sequenced and annotated [9]. It holds one of the largest bacterial genomes sequenced to date with 9.7 Mbp and 9,221 predicted open reading frames (ORFs).

**Fig. 1:**
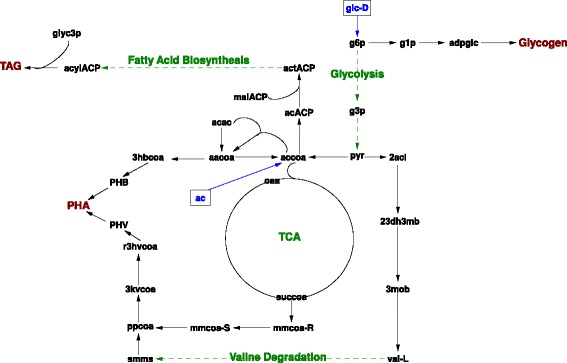
Simplified possible pathways of storage metabolism in *R. jostii* RHA1 for the three main storage compounds: glycogen, PHA, and TAG. glc-D: alpha-D-Glucose, adpglc: ADP-glucose, g1p: D-Glucose 1-phosphate, g6p: alpha-D-Glucose 6-phosphate, g3p: D-Glyceraldehyde 3-phosphate, pyr: Pyruvate, 2acl: 2-Acetolactate, 23dh3mb: 2,3-Dihydroxy-3-methylbutanoate, 3mob: 3-Methyl-2-oxobutanoic acid, val-L: L-Valine, smms: (S)-Methylmalonatesemialdehyde, accoa: Acetyl-CoA, oaa: Oxaloacetate, TCA: Tricarboxylic acid cycle, succoa: Succinyl-CoA, mmcoa-R: (R)-Methylmalonyl-CoA, mmcoa-S: (S)-Methylmalonyl-CoA, ppcoa: Propanoyl-CoA, 3kvcoa: 3-Ketovaleryl-CoA, r3hvcoa: (R)-3-Hydroxyvaleryl-CoA, PHB: Poly-β-hydroxybutyrate, PHV: Poly-3-hydroxyvalerate, PHA: Poly-β-hydroxyalkanoate, 3hbcoa: (S)-3-Hydroxybutanoyl-CoA, aacoa: Acetoacetyl-CoA, ac: Acetate, acac: Acetoacetate, TAG: Triacylglycerol, glyc3p: Glycerol 3-phosphate, acylACP: Acyl-[acyl-carrier-protein], actACP: Acetoacetyl-ACP, malACP: Malonyl-[acyl-carrier protein], acACP: Acetyl-[acyl-carrier-protein]

To face rapidly changing environmental conditions in its natural habitat, the genome of *R. jostii* RHA1 also contains many genes for the metabolism of various storage compounds including polyphosphate (polyP), glycogen, wax esters (WE), triacylglycerols (TAG), and polyhydroxyalkanoates (PHA) [10]. It was experimentally observed that, under nitrogen-limited (N-limited) condition on different substrates, *R. jostii* RHA1 accumulates these various storage compounds at the same time, but in different proportions [10]. It remains unclear how to predict the carbon fluxes to the various storage compounds. Figure 1 shows simplified possible metabolic pathways for synthesis of these storage compounds from central metabolites. Glycogen being a polymer of glucose is synthesized from a metabolite near the beginning of the glycolysis pathway. PHA (made out of poly-β-hydroxybutyrate [PHB] and poly(3-hydroxyvalerate) [PHV]) and TAG, are derived from acetyl-CoA, the metabolic point of entry of acetate. Differences in the length of the pathways between substrate (in this case, glucose and acetate) and the storage compound synthesized may suggest differences between metabolic optimality for the different compounds with respect to the mixture of storage compounds accumulated. However, other optimality principles can also be used for the predictions. It should be noted that since acidic methanolysis cleaved the acyl groups in both WE and TAG [11], the experimental data included fatty acids of both WE and TAG. Predicting TGA is also equivalent to predicting WE, since the precursors of the TAG and WE are neutral fatty acids and they are equivalent from a metabolic flux point of view. Polyphosphate (polyP) was not included in this study because the proportion of phosphorus in the biomass did not vary in the conditions tested.

To investigate how to predict the accumulation of storage compounds during nutrient-limited conditions with regard to the consumption of carbon substrate, the genome-scale metabolic network of *R. jostii* RHA1 (i.e., *i*MT1174) was reconstructed *in silico*. For this reconstruction, storage compounds were defined independently of the biomass composition as pseudo-secretory by-products; that is, although storage compounds are not secreted, they are set so that they are secreted by the metabolic model for analysis of the storage metabolism. This model definition allows the study of the variation of storage metabolism.

Genome-scale metabolic models are typically underdetermined (i.e., no unique solution exist) because the number of metabolites (mass balance equations) are lower than the number of reaction fluxes (variables). Consequently, they can be analyzed by means of linear programming to find an optimal metabolic flux profile as defined by a linear objective function (optimization criterion). Therefore, these objective functions are central to the prediction exercise. In the context of the current study, here are the two main questions we aim to answer. (1) Is there an objective function that enables us to predict the proportions of storage compound(s) and their associated metabolic fluxes observed under N-limited condition? (2) What are some of the main factors influencing the proportions of the storage compounds accumulated?

Several objective functions have been defined and examined in the past to study a large number of metabolic situations [12, 13], here are some examples. For a number of bacterial species, the maximization of the growth rate (or yield as they are not always independent) was reported to be successful at predicting experimentally observed exponential growth phenotypes [14–16]. Accounting for possible minimization of cellular “effort” in utilizing available energy and external resources, it was found that the minimization of the sum of reaction fluxes (Manhattan norm of the flux vector) was a good way to predict the outcome of the metabolic network [17], which was interpreted as a maximally efficient use of the available biochemical reactions. Finally, for predicting the metabolism of gene-deletion mutants, a successful objective function was to find the reaction flux distribution profiles most similar to the ones of the wild-type growing in the same conditions, an objective function known as minimization of metabolic adjustments (MOMA) [16].

In the current study, maximization of the growth rate (and yield) was adopted as the main objective function for the growth of RHA1 during non-limited growth condition. However, the difficulties arise for the N-limited storage accumulation because the synthesis of new biomass does not occur. Thus, novel objective functions needed to be examined, and seven objective functions were evaluated.

1. minimization of the metabolic adjustment between the N-limited and non-limited conditions (environmental MOMA; inspired by [16]),
2. minimization of the metabolic fluxes (i.e., minFluxes or flux minimization) [17],
3. minimization of ATP production rate (minATP) [13],
4. maximization of ATP production rate (maxATP) [13],
5. minimization of the production rate of redox potential (minNADH) [13],
6. maximization of the total storage fluxes in the N-limited condition (maxStorage),
7. maximization of the total storage fluxes in conjunction with environmental MOMA (maxStorage+environmental MOMA).

When environmental MOMA was used for which the non-limited and N-limited culture phases interact, the implementation of these objective functions consisted in developing an optimization algorithm by first maximizing the growth rate under non-limited conditions, and then searching the solution space thus delimited using the above mentioned objective functions.

The performance of the objective functions in predicting the accumulation of the mixture of storage compounds on two different carbon sources (glucose and acetate) under N-limited conditions were compared with our experimental observations using a Bayesian approach. Finally, ^13^C-labelling experiments to characterize fluxes during the non-limited growth phase were conducted to determine how much the reaction fluxes during the non-limited growth phases constrained the N-limited metabolic model solution compared to simply measuring the fluxes of storage compounds accumulated during the N-limited culture phase.

## Results and discussion

### Features of the RHA1 metabolic network

The reconstructed *in silico* metabolic network of *R. jostii* RHA1 (*i*MT1174) contains 1,243 balanced (intracellular) compounds, 1,935 unique reactions, and 1,174 ORFs (Table 1). Totally, it also includes 330 extracellular compounds that are associated with 518 exchange reactions. Through manual curation, 495 reactions were added to fill metabolic gaps in the network, which was justified by biochemical literature data from either *R. jostii* RHA1 or related species. Beside gap fillers, we made a special effort to include a number of xenobiotics degradation pathways (consisting of 131 transport reactions and 326 conversion reactions) in order to make the model most useful for the research community interested in *R. jostii* RHA1. Mono-, di-, tri-, and tetra-chlorobiphenyl were added in *i*MT1174 metabolic model; reactions A000540 – A00104 show all the enzymatic steps that were associated with PCB degradation; the genes involved in degradation of these PCBs were bphA, bphB, bphC, and bphD [8, 18–20].

**Table 1:** Properties of the *in silico* metabolic network of *R. jostii* RHA1 (*i*MT1174)

Property	Quantity
Pathway	114
Total reactions (unique)	3,007 (1,935)
Biochemical conversion (unique)	2,489 (1,417)
Transport	518
Reactions with ORF^a^ (% of total reactions)	1,876 (62.39 %)
Number of ORFs	1,174
Total metabolites	1,573
Intracellular	1,243330
Extracellular	330

^a^Open reading frame

Seven storage compounds were defined independently of the biomass composition as extracellular compounds connected to 11 fictional transport reactions to study the variation of storage compound accumulation namely: glycogen, α,α-trehalose, PHB, PHV, TAG, WE, and the non-carbon-based polyP. As these compounds were observed experimentally or detailed genomic investigation revealed the presence of specific genes [10], pathways for the synthesis and the degradation of these compounds were included in the biochemical model. Biomass composition of *R. jostii* RHA1 is detailed in Additional file 1, all reactions and metabolites involved in the genome-scale metabolic model of *R. jostii* RHA1 are detailed in Additional file 2, and unique reactions and metabolites involved in the genome-scale metabolic model of *R. jostii* RHA1 are detailed in Additional file 3. A specific property of the *i*MT1174 model is the higher frequency NAD, NADH, and oxygen in reactions than the energy-associated metabolites, which is different than with most published bacterial genome-scale metabolic models. This is because of the large number of oxygenase and oxidoreductase reactions involved in xenobiotics degradation pathways and included in the current *i*MT1174 model.

### Experimentally measured conversion rates

**Fig. 2:**
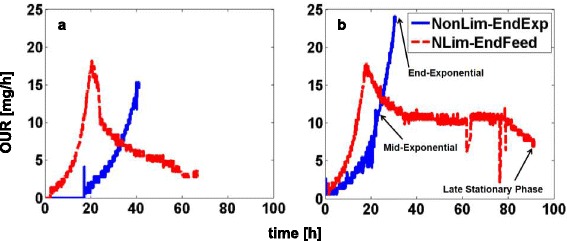
The oxygen uptake rate time profile of RHA1 growth on **a** glucose and **b** acetate in the non- and N-limited culture conditions. Three samples were collected at the middle and end of the exponential growth phase in the non-limited condition and at the late stationary phase in the N-limited condition

The conversion rates of various storage compounds when glucose and acetate were used as the sole carbon and energy sources during non-limited and N-limited conditions were experimentally measured. Initial ammonium concentrations in the N-limited cultures were adjusted such that the N-source was 10 times lower than that in the non-limited cultures; the nitrogen sources in the N-limited cultures were exhausted at a time equivalent to the mid-exponential phase of the non-limited cultures. Oxygen uptake rate (OUR) time profiles of RHA1 in culture media containing glucose or acetate and either non-limiting or limiting concentrations of ammonium demonstrated a clear change in the respiration behaviour of the culture when the nitrogen source was exhausted (Fig. 2). Samples to determine the composition of the non-limited cultures were obtained at the middle and the end of the exponential phase, while the composition of the N-limited cultures was based on samples obtained immediately before the ammonium was completely consumed and late stationary phase at which the respiration rate became very low (Fig. 2).

**Fig. 3:**
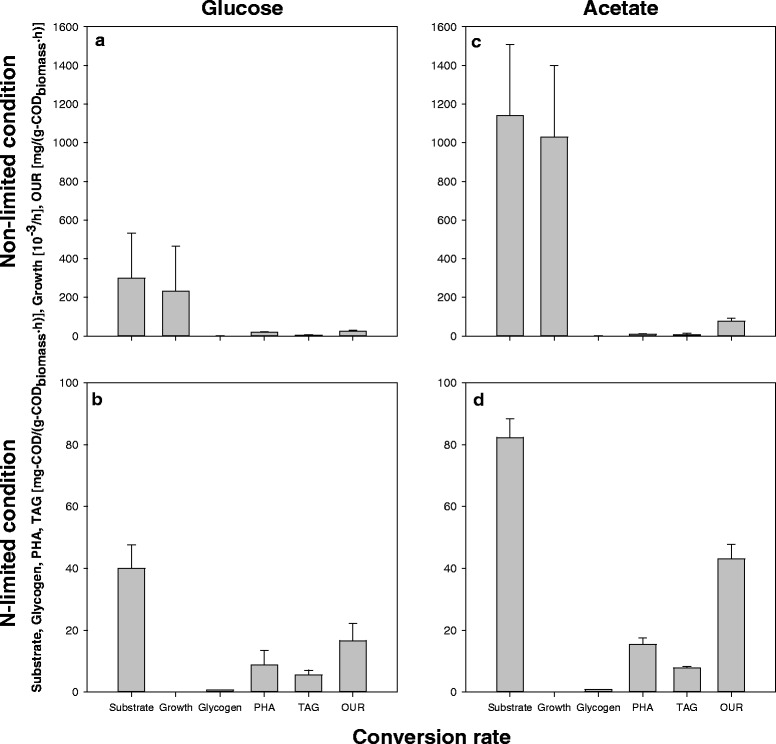
The conversion rates of storage, substrate, and oxygen, and the growth rate of *R. jostii* RHA1 on glucose (**a** and **b**) and acetate (**c** and **d**) in the non- and N-limited conditions. Panels a and c represent the conversion rates in the non-limited condition, while panels b and d show those in the N-limited condition. Note that the conversion rate of PHA is the sum of those of PHB and PHV

Microbial activities in the non-limited and N-limited cultures were determined by monitoring the concentrations of several compounds over time: substrate (either glucose or acetate, concentrations determined by enzymatic assays and by chemical oxygen demand [COD]), NH_4_^+^, PO_4_^3−^, glycogen, PHB, TAG. Additionally, the consumption rate of O_2_ was also monitored over time. Using these data, conversion rates were calculated. Considering the elemental and COD balances, the conversion rates were reconciled (i.e., adjusted for the balances to close) and their statistical consistency checked (i.e., tested for the presence of gross measurement errors) [21]. The measured conversion rates and the results of the reconciliation procedure are presented in Additional file 4. In these experiments, *R. jostii* RHA1 consumed acetate faster than glucose, and the specific substrate uptake rate during the N-limited phase was less than 14 % of the one in the non-limited phase (Fig. 3). During the N-limited phase, the glucose culture stored 58 % of the substrate COD consumed, with 74 % of it being stored as PHA and 23 % as TAG (Fig. 3). Finally, for the same culture phase, the acetate culture stored 48 % of the substrate COD consumed, with 78 % in the form of PHA and 20 % in the form of TAG.

### Metabolic model of storage accumulation

Accumulations of storage compounds by RHA1 were examined using the genome-scale metabolic model (*i*MT1174) and comparing seven objective functions. To do so, a list of unique reactions was generated by eliminating the redundancy in the network due to the large number of isoenzymes (i.e., the model used 1,935 unique reactions, see Additional file 3). The goal was to determine the most suitable objective function to predict the biosynthesis rate of glycogen, PHA, and TAG after the exhaustion of ammonium. For the purpose of this paper, PHA was assumed to be a co-polymer of PHB and PHV in the same proportion as experimentally observed by [10]. It should be noted that since acidic methanolysis cleaved the acyl groups in both WE and TAG [11], the experimental data included fatty acids of both WE and TAG. Predicting TAG is also equivalent to predicting WE, since the precursors of the TAG and WE are neutral fatty acids and they are equivalent from a metabolic flux point of view.

**Fig. 4:**
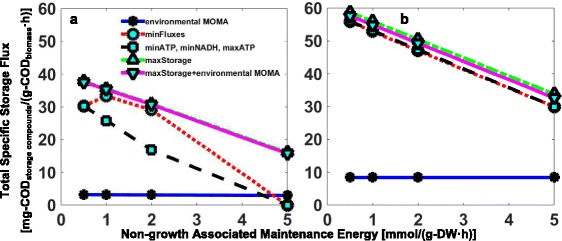
Total storage flux as a function of the non-growth-associated maintenance energy on **a** glucose and **b** acetate. Objective functions are: environmental MOMA, minimization of fluxes, minimization and maximization of ATP production, minimization of NADH production, maximization of the storage pools, and maximization of the storage pools along with environmental MOMA

The total specific storage production rate (defined as total mg-COD_storage compounds_/(g-COD_biomass_ · h)) was found to be very sensitive to the non-growth associated maintenance energy (NGAM) for most of the objective functions examined as was expected (Fig. 4). However, variations in NGAM did not affect the observed orders in the yields of storage compounds obtained for all objective functions. To obtain the appropriate NGAM for each objective function such that the simulated total storage production rates (in COD units) were the same as those obtained experimentally, a different NGAM was determined for each objective function. Doing so assured that the relative production rate of each storage compound and not the total production rate was the basis to differentiate the applicability of each objective function. The total storage fluxes for the environmental MOMA objective function alone were insensitive to NGAM, and they remained much lower than the experimentally measured ones (Fig. 4). This was due to the generation of futile cycles in the simulation, which were dissipating the carbon and energy. Nonetheless, a NGAM value of 0.5 mmol/(g-DW·h) was adopted for the environmental MOMA objective function for comparison purposes.

**Fig. 5:**
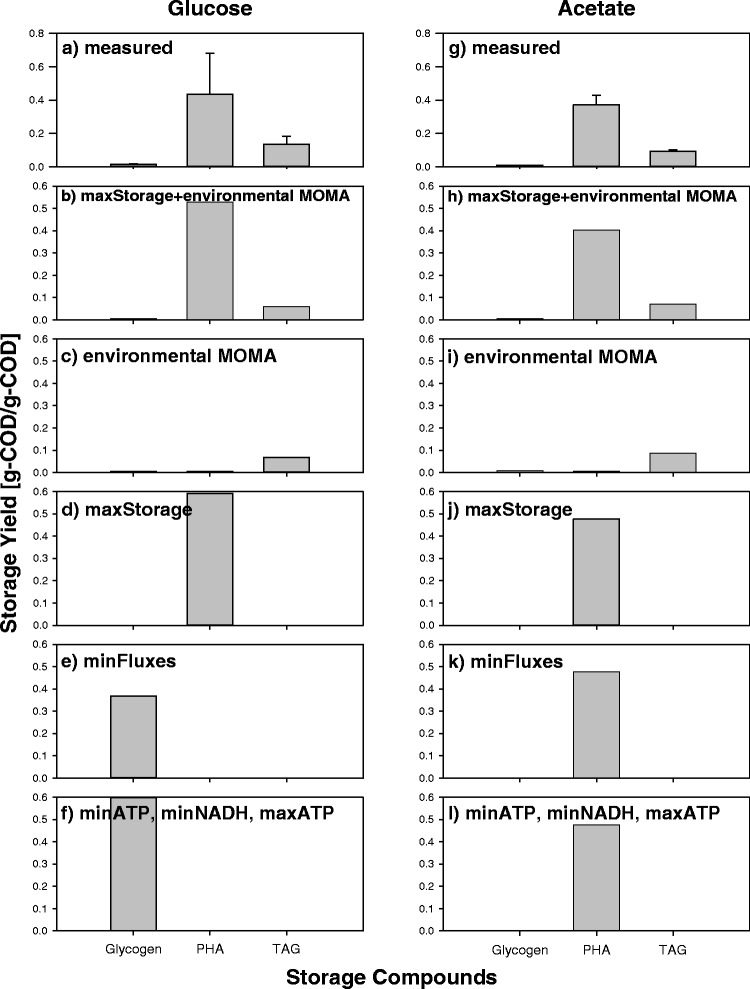
Measured and calculated storage yields of three different storage compounds glycogen, PHA and TAG on glucose (**a**–**f**) and acetate (**g**–**l**). **a** measured storage yields on glucose, along with the error bars, **b** maximization of the storage fluxes+environmental MOMA on glucose, **c** environmental MOMA on glucose, **d** maximization of the storage fluxes on glucose, **e** minimization of the metabolic fluxes on glucose, **f** minimization and maximization of ATP production, and minimization of NADH production on glucose, **g** measured storage yield on acetate, along with the error bars, **h** maximization of the storage fluxes+environmental MOMA on acetate, **i** environmental MOMA on acetate, **j** maximization of the storage fluxes on acetate, **k** minimization of the metabolic fluxes on acetate, **l** minimization and maximization of ATP production, and minimization of NADH production on acetate. Note that the storage yields were identical for minimization and maximization of ATP production and minimization of NADH production; therefore, they were grouped in one graph

For the seven objective functions compared, two patterns of storage accumulation were simulated during the period of N-limitation and carbon excess; most of the objective functions simulated a single storage compound accumulation, while objective functions based on the environmental MOMA (environmental MOMA alone and maxStorage+environmental MOMA) predicted the accumulation of a mixture of storage compounds (Fig. 5b,c,h,i).

For the objective functions that simulated a single storage compound accumulation, only the maxStorage objective function simulated PHA accumulation when the culture contained glucose and acetate as C-source (Fig. 5d,j). The other objective function in this group simulated the accumulation of glycogen and PHA, respectively, when glucose and acetate were the C-sources (Fig. 5e,f,k,l). These objective functions are subsets of the family of objective functions aiming at the minimization of the reaction fluxes, an approach justified by the principle that microbes aim at maximizing their metabolic efficiency. Thus, it seems that the strict maximization of metabolic efficiency is not the main factor determining the relative composition of the mixture of storage compounds accumulated after the sudden onset of nitrogen limitation.

Using a Bayesian-based objective function discrimination method [13], all the information in Fig. 5 were combined to evaluate the relative probability of each objective function for the prediction of the mixture of storage compounds in the N-limited culture phase. Maximization of storage in conjunction with environmental MOMA was the most probable objective function (posterior probability share of 76.2 %), and the maximization of storage alone was the second most probable one (posterior probability share of 21.3 %) (Table 2). All the other objective functions had posterior probability shares lower than 2.5 %.

**Table 2:** Posterior probability share of each objective function, listing in descending order of probability

Objective Function^a^	Posterior Probability Share (%)
Maximization of storage and environmental MOMA	76.20
Maximization of storage	21.30
Environmental MOMA	2.37
Flux minimization	6.57 × 10^−2^
Minimization of ATP production	2.13 × 10^−2^
Maximization of ATP production	2.13 × 10^−2^
Minimization of NADH production	2.13 × 10^−2^

^a^Note that the experimentally observed NGAM were 23 and 39 mg-COD_storage compounds_/(g-COD_biomass_·h) on glucose and acetate, respectively. The calculated NGAM values (in mmol/(g-DW·h)) at the measured total storage flux for different objective functions on glucose are as follows: minFluxes: 2.236, maxATP, minATP, minNADH: 1.200, maxStorage: 3.502, and maxStorage+environmental MOMA: 3.44. The calculated NGAM values (in mmol/(g-DW·h)) at the measured total storage flux for different objective functions on acetate are as follows: minFluxes: 3.385, maxATP, minATP, minNADH: 3.409, maxStorage: 4.027, and maxStorage+environmental MOMA: 3.823

The simulation results presented here could be understood as possible outcomes of global regulatory principles applied by *R. jostii* RHA1 when faced with sudden nutrient-limited condition. Based on our results, it appears that the cells maintain the reaction flux profiles under carbon-excess/nutrient-limited conditions somewhat similar to the one developed during the non-limited growth conditions while maximizing their storage accumulation. This seems logical from a global regulation perspective as this solution would simply follow the regulatory program for growth and diverting the excess carbon found locally in the biochemical network to the closest storage compound. It would also maximize the responsiveness of the organism, while minimizing the protein turnover that would be necessary to adapt to the new conditions.

### Effect of biomass composition on simulated storage mixture

The previous section concluded that the metabolic fluxes developed during the non-limited growth determined the fluxes during the N-limited storage accumulation period. Consequently, changing the biomass composition could affect the relative composition of the predicted mixture of storage compounds. This hypothesis was tested by substituting the biomass of *R. jostii* RHA1 used in our model by the *E. coli* biomass of the *i*JR904 [22] and *i*AF1260 [23] metabolic models. Note that Additional file 5 compares the biomass composition of three metabolic models *i*MT1174, *i*JR904, and *i*AF1260. The most probable objective function (maxStorage+environmental MOMA) was used for these simulations.

**Fig. 6:**
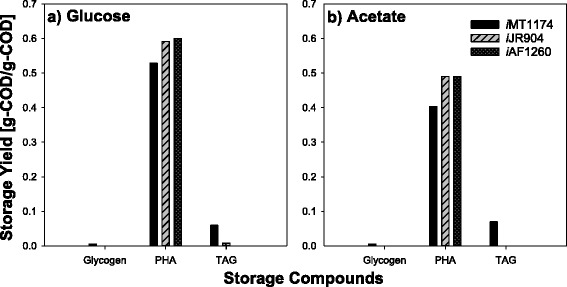
Calculated storage yields of three different storage compounds (glycogen, PHA, and TAG) simulated by the *R jostii i*MT1174 model for glucose **a** and acetate **b** consumption using either *i*MT1174 or *E. coli* biomass definitions. Two *E. coli* biomass definitions were used: *i*JR904 and *i*AF1260 models. For these simulations, the objective function was maxStorage+environmental MOMA

Using the *E. coli* biomass of the *i*JR904 model with both carbon substrates led to an increase in the proportion of PHA within the storage compounds accumulated by 11.9 % on glucose and 21.6 % on acetate, while the proportions of TAG and glycogen both decreased (Fig. 6). Using the *E. coli* biomass of the *i*AF1260 model with both carbon substrates yielded similar results. The decrease in TAG could be attributed to the lower lipid content in the *E. coli* biomass, but it is not clear why the proportion of glycogen would also decrease. This simulation study showed that the quantitative predictions of storage compound accumulation depends on the biomass composition, as it was found for other metabolic fluxes in other studies [24].

### Constraints imposed by storage accumulation rates vs. environmental MOMA

**Fig. 7:**
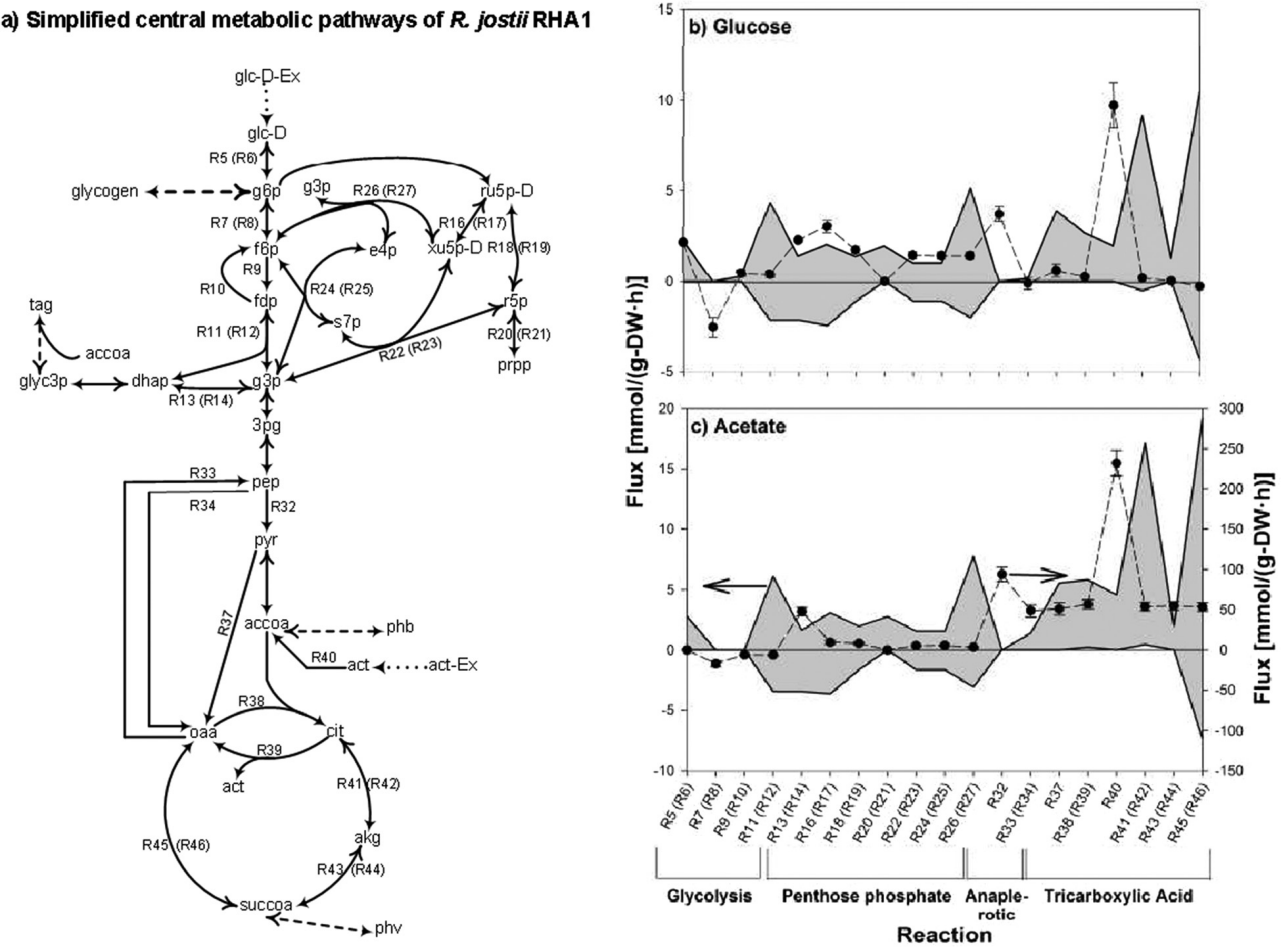
Comparison of the fluxes of 19 central metabolic reactions of the *R. jostii* RHA1 network **a** estimated using ^13^C-MFA for non-limited growth phase and using FBA for N-limited phase with glucose **b** or acetate **c** as substrate. For the non-limited phases, fluxes were calculated using the ^13^C-MFA approach. The data points (circle) reported are the average of 100 solutions inversely weighted on the fitting error of the ^13^C-MFA model; the error bars are Cochran’s weighted standard errors [48]. For the N-limited phases, the ranges of fluxes (shadow between maximum and minimum) were computed using the genome-scale FBA at zero nitrogen uptake rate. Note that the *y*-axis scales for panels b and c are not the same, and that the *y*-axis of non-limited data and N-limited ranges of fluxes are not the same in panel c. The *x*-axis includes the reaction IDs appeared in the simplified central metabolic network of *R. jostii* RHA (panel a). The corresponding KEGG IDs can be found with the complete central metabolic network in Additional file 6. The reactions in parentheses are the backward reactions of the reversible reactions

The MOMA objective function was introduced to the flux balance analysis to compare the wild-type and mutant organisms [16]; however, in this study we extended the application of this objective function to compare balanced fluxes of metabolisms operating two different but successive environmental conditions: non-limited and N-limited conditions. In order to understand better the constraints imposed on the N-limited metabolic flux solution by the environmental MOMA, we first determined by FBA the range of fluxes through reactions of the central metabolism on the substrate and nutrient uptake rates and storage compound accumulation rates were fixed according to experimental measurements (shadow represents range of possible fluxes between minimum and maximum in Fig. 7b and c). This range was then compared with fluxes through the same reactions during the non-limited growth phases as determined by ^13^C-metabolic flux analysis (^13^C-MFA) to elucidate the metabolic behaviour of the *R. jostii* RHA1 cell (its central metabolic network was depicted in Additional file 6; Fig. 7b and c); it was not possible to determine the fluxes during the N-limited phase because amino-acids are not synthesized during this phase due to the nitrogen limitation. As it can be seen, the range of the reaction fluxes during the N-limited culture phase remain quite wide, with the net fluxes of certain reactions being possibly in either direction (i.e., the range can be both positive and negative; Fig. 7). Therefore, the environmental MOMA objective function imposes a more specific flux solution than the possible range.

## Conclusions

*R. jostii* RHA1 can accumulate several storage compounds simultaneously, but the approach to predict the relative synthesis rates of these compounds during nutrient-limited conditions remain to be clarified. A genome-scale metabolic model of *R. jostii* RHA1 (*i*MT1174) was built, and used in flux balance analysis with different objective functions to predict the fluxes of storage compounds during a period of N-limitation following a period of balanced maximal growth. Among the seven objective functions examined, the objective function maximization of storage in the N-limited condition combined with environmental MOMA was able to reproduce best the storage accumulation fluxes observed experimentally for glucose and acetate as C-source. To further understand the constraints imposed by the environmental MOMA objective function, the possible variability of fluxes through 19 central metabolic reactions during N-limited storage-accumulating conditions were compared to fluxes through the same reactions during non-limited growth obtained by ^13^C-MFA. The comparison showed how the environmental MOMA could constrain the reaction fluxes in the N-limited conditions. Altogether, the data presented in herein suggest that for *R. jostii* RHA1 during N-limited storage accumulating conditions the storage production to efficiently save the C-source, while the reaction fluxes remain somewhat similar to the profile in the immediately preceding period when its growth was not limited. Finally, the analysis demonstrates the applicability of the MOMA objective function to analyze rapid changes in successive different environmental conditions such as non-limited growth followed by N-limited storage-accumulating substrate consumption.

## Methods

### Highlights of the model reconstruction procedure

As the first step in reconstructing the cellular metabolic network of *R. jostii* RHA1, the list of genes in its annotated genome sequence was converted to a list of associated balanced biochemical reactions, which form the basic framework of the model. Subsequently, the model was analyzed in light of literature data to determine if some reactions may have been missing from the list of annotated genes. The reactions were added along with transport reactions to make a physiologically meaningful cellular model. In the case of RHA1, although annotated, several degradation pathways of various xenobiotics were missing from the Kyoto Encyclopaedia of Genes and Genomes (KEGG) (the primary biochemical database used [25]) and they were added to the model using literature data and other online databases [26, 27] to account for its catabolic ability. Then, the metabolic network was completed by the pathways of seven storage compounds that have also been considered and manually curated in the model: glycogen, α,α-trehalose, PHA such as PHB and PHV [10, 28], TAG [10, 29, 30], and polyP. Finally, eight macromolecules were assigned to make up the biomass (e.g., protein, DNA, RNA, phospholipids, small molecules such as electron carriers and coenzymes, peptidoglycan, carbohydrates, and corynomycolic acids). We also figured out the directionality and reversibility of most of the reactions in the *i*MT1174 model using online databases [31, 32]. The non-growth-associated maintenance energy (NGAM) was set differently for different objective functions to adjust the total flux of storage compounds (mg-COD_storage compounds_/(g-COD_biomass_·h)) to values comparable to those of the experimental data. Finally, the growth-associated maintenance energy (GAM) was set to 30 mmol-ATP/g-DW according to the literature data [24].

Additional file 1 details the biomass and storage lipid compositions. Additional files 2 and 3 also provide the complete lists of genes, enzymes, reactions, and metabolites appear in the network of RHA1. Additional file 2 shows all metabolites and reactions involved in specified biochemical pathways, along with added reactions; it contains reaction properties such as name, ID, definition, equation, directionality, lower and upper bounds, enzyme commission (EC) numbers, ORFs, replaced reactions, and references. Additional file 3 summarizes unique reactions and metabolites, along with their properties, that were simulated in this work. This biochemical network was converted into a stoichiometric matrix (**S**) with rows and columns corresponding to the metabolites and reactions, respectively, in order to be quantified by means of the FBA technique.

### Flux balance analysis

The gene-protein-reaction (GPR) association, along with the stoichiometric matrix, is a qualitative representation of the cellular network. The metabolic model of RHA1 was studied quantitatively by setting up a series of mass balances around each intracellular metabolite that can be expressed in matrix notation as: $\frac{d\mathrm{X}}{dt}=\mathbf{S}.\mathbf{v}$ where **X** is the (m × 1) vector of the concentrations of the balanced metabolites (i.e., intracellular and macromolecular compounds), **v** denotes the (n × 1) vector of the entire metabolic fluxes, and **S** stands for the m × n stoichiometric matrix; note that in the case of the *i*MT1174 model with the unique reactions, the dimension of the stoichiometric matrix is 1243 × 1935.

The model was basically solved by assuming that the cellular network is at steady-state, which simplifies the previous equation to **S.v** = **0**. This assumption is plausible due to the fast equilibration of intracellular metabolite pools (time-scale of seconds) compared to the time-scale of genetic regulation (minutes) [16] and due to high reaction rates of intracellular reactions compared to phenotypic exchange rates such as substrate uptake, cell growth, and by-product secretion rates [33–35]. Consequently, the output of the model is a distribution of metabolic fluxes through the various chemical reactions (note that the kinetics is not modeled). Since the number of fluxes normally exceeds the number of metabolites (n > m), the problem is said to be underdetermined. In this case, there is a solution space for the fluxes that can be studied by optimizing proper objective functions subject to defined constraints (physicochemical constraints as mass balance and enzymatic capacity constraints as inequalities) [36].

The goal of the study was to examine objective functions for the quantitative prediction of the accumulation rates of storage compounds during conditions of nitrogen-limitation and carbon-excess using the *i*MT1174 metabolic network. Seven objective functions were implemented namely: 1) minimization of the metabolic adjustment between non-limited growth and N-limited storage accumulation (environmental MOMA), 2) minimization of the metabolic fluxes (i.e., minFluxes) [17], 3) minimization of ATP production rate (minATP) [13], 4) maximization of ATP production rate (maxATP) [13], 5) minimization of the production rate of redox potential (NADH) (e.g., minNADH) [13], 6) maximization of the total storage fluxes in the N-limited condition (maxStorage), and 7) maximization of the total storage fluxes in conjunction with environmental MOMA (maxStorage+environmental MOMA). When the metabolic fluxes during the non-limited growth phase needed to be simulated (i.e., with the objective function using the environmental MOMA approach), the maximization of growth rate (and yield) was adopted.

We solved the linear programming problem using the COBRA toolbox [36] within *The Language of Technical Computing* MATLAB environment (Mathworks Inc, Massachusetts). The model was balanced for elemental mass and charges and a number of reactions were considered irreversible (physicochemical constraints), but the enzymatic capacity of reactions remained unbounded. The substrate uptake rate (in the non-limited and N-limited conditions), phosphorus and ammonium uptake rates in the non-limited condition were constrained according to our experimental observations. N-limited conditions were then simulated by reducing to zero the uptake flux of NH_3_ into the cellular network.

### Experimental procedures on non- and N-limited culture conditions of *R. jostii* RHA1

To validate the model we performed two different sets of culture conditions namely: N- and non-limited conditions of *R. jostii* RHA1 in batch; labelled and unlabelled glucose and acetate were employed as the sole carbon sources. The labelling tests were performed using a μ-24 bioreactor (Applikon Biotechnology), while the unlabelled batch tests were accomplished with a respirometer model AER-200 (Challenge Technology).

### Bacterial strain and growth conditions

*R. jostii* strain RHA1 was cultivated aerobically at 28 °C and pH 7 in mineral salts medium (MSM) according to Schlegel et al. [37]. The MSM medium contains: NH_4_Cl (1.00 g/L), KH_2_PO_4_ (1.50 g/L), Na_2_HPO_4_.12H_2_O (9 g/L), FeNH_4_-Citrate (1.20 mg/L), MgSO_4_.7H_2_O (0.20 g/L), CaCl_2_ (0.02 g/L), Hoagland solution (2.00 mL/L), and carbon source (10 g-COD/L). Glucose and sodium acetate were used as the sole carbon sources. Cells were harvested during mid-exponential (non-limited condition) and end-exponential (both non- and N-limited conditions), and late stationary phases (N-limited condition), centrifuged and stored at −80 °C for further analyses. To generate nitrogen-limiting conditions, the concentration of ammonium chloride in MSM was reduced to 0.1 g/L such that the nitrogen source was exhausted before the carbon source.

### Biochemical analysis

Total and volatile suspended solids (TSS/VSS) were measured according to the standard method 2540 D/2540 E [38]; chemical oxygen demand (COD) was quantified according to the standard method 5220 D [38]; phosphorus was measured by the Ascorbic Acid method (standard methods 4500-P E) [38]; ammonium concentration was measured by colorimetry [39] (spectrophotometric measurements at 630 nm); cellular glycogen was quantified by a hexokinase enzymatic kit and colorimetry (Hexokinase protocol, measurement of NADH concentration at 340 nm, Sigma, St. Louis, MO) [40]; cellular PHA was measured by colorimetry [41, 42]; (spectrophotometric measurements at 235 nm). Spectrophotometric measurements of the last four components were performed in microplates using a SpectraMax5 reader (Molecular Devices, LLC, USA).

Cellular TAG was measured by gas chromatography (GC) according to [43] with some modifications as follows. For the identification of lipids, cultures were harvested by centrifugation at 6000 g for 15 min and lyophilisation overnight. 5-8 mg of lyophilized cells were resuspended in 0.6 mL of chloroform and 0.6 mL of methanol containing 15 %(v/v) H_2_SO_4_; 1 μL Trinonadecanoin (Nu-Check Prep Inc., Elysian, MN) was used as internal standard for quantification [44]. Methanolysis was carried out at 100 °C for 2.5 h. After cooling to room temperature and then on ice, 0.3 mL of deionized water was added to the solution, which was then vigorously vortexed for 1 min. After phase separation, 0.45 mL of the organic phase (bottom layer) was removed and transferred to a small screw-cap glass vial [43]. The organic phase containing fatty acid methyl esters (FAMEs) was analyzed by using an Agilent 6890 N GC system equipped with an Agilent HP-88 column (60 m by 0.25 mm, 0.2 μm thick film) with helium as the carrier gas at flow rate of 2 mL/min. A 1 μL portion of the organic phase was injected with a 50:1 split ratio using the auto-sampler. The inlet was maintained at 250 °C. The oven was held at 175 °C for 15 min, heated to 220 °C at 3 °C/min, and then held at 220 °C for 5 min. Peak detection was performed by a flame ionization detector, which was maintained at 280 °C. The fatty acids were identified and quantified by comparison to standard FAMEs (Sigma). Fatty acid content was defined as the percentage of the ratio of fatty acids to cell dry weight (% CDW).

### Analysis of labelled amino acids and ^13^C-metabolic flux analysis

Fresh *R. jostii* RHA1 culture, which was cultivated aerobically at 28 °C and pH 7 in MSM, was transferred (0.5 mL) into 5-mL cells of the μ-24 bioreactor (Applikon Biotechnology); these reactor cells had already been filled with 4.5 mL of fresh MSM supplemented with labelled carbon sources including 1-^13^C-Acetate and 2-^13^C-Acetate Sodium salt, and 1-^13^C-Glucose as the sole carbon sources; and then, cells were harvested during the exponential growth phase. It should be noted that the culture condition was at the non-limited growth condition (i.e., 1.00 g/L of NH_4_Cl).

Proteinogenic amino acids of the biomass were analyzed using gas chromatography mass spectrometry (GC-MS) technique according to Nanchen et al. with some modifications [45]. The biomass samples which had already been stored at −80 °C were thawed and resuspended in 1.5 mL sterile deionized water, vortexed, and homogenized using ultrasonic treatment (60 watts) for 15 min. After resuspension, 100 μL of sample was transferred into a 2 mL microcentrifuge tube, further spun down the cell pellets (at 15800 g at room temperature for 15 min). Cell pellets were washed twice by resuspension in 1 mL 0.9 % NaCl, and centrifuged at 15800 g at room temperature for 15 min. The washed pellets were resuspended in 1 mL of 6 M HCl, and hydrolyzed for 24 h at 110 °C in a well-sealed screw-capped tube to prevent evaporation. The hydrolyzate was dried overnight in a heating block at 70 °C and under a constant air stream in a fume hood. The dry hydrolyzate was dissolved in 30 μL of a reagent containing 10 mg of Methoxyamine Hydrochloride per 1 mL of Anhydrous Pyridine. 1 μL of the internal standard was added into each sample tube, note that the internal standard was 750 ng/μL of deutrated myristic acid (so-called D_27_-myristic acid), the samples were mixed by vortex and sonication for several times, each time taking 10–20 s. Furthermore, the samples were centrifuged at 15000 rpm for 10 min at room temperature. The samples were transferred into GC-MS vials and cooked at 70 °C for 30 min in a hot block. 70 μL of N-tert-butyldimethylsilyl-N-methyltrifluoroacetamide (TBDMS) was added into the vials and cooked at 70 °C for 1 h in a hot block to derivatize the samples. GC-MS analyses were performed with an Agilent 5975C mass selective detector coupled to a 7890A gas chromatograph (Agilent Technologies, Santa Clara, CA, USA) fitted with a 7693 autosampler and a DB-5MS+DG capillary column (30 m plus 10 m Duraguard^®^), diameter 0.25 mm, film thickness 0.25 mm (Agilent J &W, Santa Clara, CA, USA). The GC temperature program started with a 1 min hold at 60 °C followed by a 10 °C/min ramp to 300 °C. Bake-out was at 320 °C for 10 min. The injector and interface to the MS were held at 285 °C. The helium carrier flow rate was held constant at 1.5 mL/min (or a flow rate such that the TBDMS derivative of the D_27_-myristic acid has a retention time of 18 min). When operated in full scan mode, the scan range was 50–700 Da. 1 μL of the sample was injected in splitless mode.

We aim at estimating the reactions involved in central metabolism in the non-limited condition using ^13^C-metabolic flux analysis (^13^C-MFA). The model represents the central metabolism which includes glycolysis and gluconeogenesis, Entner-Doudoroff pathway, tricarboxylic acid (TCA) cycle, pentose phosphate pathway, anaplerotic carboxylation and decarboxylation, storage metabolic reactions, amino acid biosynthetic reactions, and anabolic routes into biomass. Additional file 6 shows the central metabolic pathways of *R. jostii* RHA1 for which ^13^C-MFA was implemented. We used the openFLUX software application under MATLAB environment (Mathworks Inc, Massachusetts) to solve for the fluxes [46]. The application is based on the Elementary Metabolite Unit (EMU) framework. Stoichiometric data on growth, substrate uptake rate, storage formation, and on the cellular composition of *R. jostii* RHA1 together with mass isotopomer distribution data of the labelled amino acids that were produced using the *iMS2Flux* software [47] were used as model input.

Weighted average of 100 solutions of central metabolic fluxes of *R. jostii* RHA1 and their respective standard errors in the non-limited condition were calculated according to [48]. Theses fluxes were compared with the corresponding minimum and maximum fluxes in the N-limited condition predicted with the FBA approach.

### Availability of supporting data

All the supporting data are included in the following additional files.

## Additional files

Additional file 1:
**Biomass and storage lipid compositions.** It includes the compositions of the RHA1 biomass, macromolecules, PHA, TAG, and WE used in this study. (XLS 64 kb) Additional file 2:
**Total metabolites and reactions and their corresponding biochemical pathways, along with exchange reactions, that are involved in the** ***i*** **MT1174 network.** It contains reaction properties such as name, ID, definition, equation, directionality, lower and upper bounds, enzyme commission (EC) numbers, open reading frames (ORFs), KEGG IDs of reactions that were replaced by added reactions (replaced reactions), and references ***(Additional file 2-1)***. It also contains unique metabolites ***(Additional file 2-2)***. (XLSX 325 kb) Additional file 3:
**Unique reactions and metabolites, along with their properties, that are involved in the** ***i*** **MT1174 network.** It includes unique reactions **(** ***Additional file 3-1*** **)** **and unique metabolites** **(** ***Additional file 3-2*** **).** These data were utilized to make the stoichiometric matrix of the *i*MT1174 metabolic network. Note also that the names, IDs and chemical formulas of the metabolites are reported. In this file, the comma separated genes were meant to show the association of the cluster of the genes encoding the respective enzyme. Moreover, some reactions are associated with more than one enzyme; in this case, the additional enzymes were placed in the subsequent rows, along with their respective genes. (XLSX 265 kb) Additional file 4:
**Elemental composition matrix and measured and reconciled conversion rates of** ***R. jostii*** **RHA1 on glucose and acetate in the non- and N-limited conditions.** (PDF 152 kb) Additional file 5:
**Biomass composition of three metabolic models** ***i*** **MT1174,** ***i*** **JR904, and** ***i*** **AF1260.** (PDF 76 kb) Additional file 6:
**Central metabolic pathways of** ***R. jostii*** **RHA1.** (PDF 329 kb)
