# Supplementary material for: Genome-scale metabolic model of Rhodococcus jostii RHA1 (iMT1174) to study the accumulation of storage compounds during nitrogen-limited condition
Source: BMC Syst Biol. 2015 Aug 7;9:43. doi: 10.1186/s12918-015-0190-y (PMC4528721; doi:10.1186/s12918-015-0190-y)
Supplement: Additional file 4: — Elemental composition matrix and measured and reconciled conversion rates of R. jostii RHA1 on glucose and acetate in the non- and N-limited conditions. (PDF 152 kb) [file 12918_2015_190_MOESM4_ESM.pdf]

Additional File 4. Elemental composition matrix and measured and reconciled conversion rates of *R. jostii* RHA1 on glucose and acetate in the non- and N-limited conditions.

Table AF4-1 – Elemental composition matrix in the non-limited growth condition

Conversion	C[g]	COD	N[h]	P[i]	COD_{total}
**Substrate**	0.38	1	0	0	1
**COD[a]**	0	0	0	0	−1
**NH_4^+**	0	0	1	0	0
**PO_4^{3-}**	0	0	0	1	0
**μ[b]**	0.32	1	0.07	0.01	0
**Glycogen**	0.38	1	0	0	0
**PHB[c]**	0.33	1	0	0	0
**PHV[d]**	0.31	1	0	0	0
**TAG[e]**	0.27	1	0	0	0
**OUR[f]**	0	−1	0	0	0
**CO_2**	1	0	0	0	0

[a]: Chemical oxygen demand; [b]: growth rate; [c]: poly-β-hydroxybutyrate; [d]: poly(3-hydroxyvalerate); [e]: triacylglycerol; [f]: oxygen uptake rate; [g]: carbon; [h]: nitrogen; [i]: phosphorus.

Note that, in the N-limited condition the columns corresponding to N and P, along with the rows corresponding to NH_4^+, PO_4^{3-}, and μ are absent.

Table AF4-2 – Conversion rates on glucose and acetate as the sole carbon sources in the non- and N-limited conditions

Conversion	Units	Glucose Non-LIMITED		Glucose N-LIMITED		Acetate Non-LIMITED		Acetate N-LIMITED	
		Measured	Reconciled	Measured	Reconciled	Measured	Reconciled	Measured	Reconciled
**Substrate**	mg-COD/(g-$COD_{biomass}$·h)	−294±236	−299±234	−28.0±12.3	−40.0±7.64	−1140±368	−1140±368	−68.4±15.8	−82.3±6.05
**COD**[a]	mg-COD/(g-$COD_{biomass}$·h)	ND[h]	−299±234	−44.2±13.6	−40.0±7.64	ND	−1140±368	ND	−82.3±6.05
**NH_4^+**	mg-N/(g-$COD_{biomass}$·h)	ND	−17.0±17.2	ND	ND	ND	−76.0±27.1	ND	ND
**PO_4^{3-}**	mg-P/(g-$COD_{biomass}$·h)	−10.0±27.9	−2.61±2.65	ND	ND	−5.50±3.50	−5.50±3.50	ND	ND
**μ**[b]	10^{-3}/h	ND	231±235	ND	ND	ND	1030±369	ND	ND
**Glycogen**	mg-COD/(g-$COD_{biomass}$·h)	1.53±0.42	1.50±0.40	0.60±0.20	0.60±0.00	1.70±0.80	1.70±0.80	0.80±0.20	0.80±0.00
**PHB**[c]	mg-COD/(g-$COD_{biomass}$·h)	11.3±3.40	11.3±3.40	7.80±4.90	6.45±4.68	5.50±1.80	5.50±1.80	9.50±1.70	9.34±1.69
**PHV**[d]	mg-COD/(g-$COD_{biomass}$·h)	26.4±7.90	26.4±7.90	18.3±11.5	10.9±8.33	12.8±4.30	12.8±4.30	22.2±4.10	21.3±3.98
**TAG**[e]	mg-COD/(g-$COD_{biomass}$·h)	3.70±2.30	3.70±2.30	5.60±1.60	5.46±1.59	7.60±5.60	7.60±5.60	7.80±0.40	7.79±0.40
**OUR**[f]	mg/(g-$COD_{biomass}$·h)	−25.3±5.70	−25.3±5.70	−18.6±6.00	−16.6±5.60	−76.0±15.3	−76.0±15.3	−44.4±4.80	−43.1±4.61
**CO_2**	mg-C/(g-$COD_{biomass}$·h)	ND	24.8±13.1	ND	7.76±1.93	ND	87.5±20.9	ND	18.7±1.73
**χ^2 Statistics**			6.63		9.21		6.63		6.63
**DOR**[g]			1		2		1		1
**h**			0.07		1.66		0.00		0.91
**Probability**			99%		99%		99%		99%

[a]: Chemical oxygen demand; [b]: growth rate; [c]: poly-β-hydroxybutyrate; [d]: poly(3-hydroxyvalerate); [e]: triacylglycerol; [f]: oxygen uptake rate; [g]: degree of redundancy; [h]: not determined. For the purpose of this paper, PHA was assumed to be a co-polymer of PHB and PHV in the same proportion as experimentally observed by [1].

References

1. Hernandez MA, Mohn WW, Martinez E, Rost E, Alvarez AF, Alvarez HM: **Biosynthesis of storage compounds by *Rhodococcus jostii* RHA1 and global identification of genes involved in their metabolism**. *BMC Genomics* 2008, **9**(600):1-13.
