# Supplementary material for: Genome-scale metabolic model of Rhodococcus jostii RHA1 (iMT1174) to study the accumulation of storage compounds during nitrogen-limited condition
Source: BMC Syst Biol. 2015 Aug 7;9:43. doi: 10.1186/s12918-015-0190-y (PMC4528721; doi:10.1186/s12918-015-0190-y)
Supplement: Additional file 5: — Biomass composition of three metabolic models i MT1174, i JR904, and i AF1260. (PDF 76 kb) [file 12918_2015_190_MOESM5_ESM.pdf]

Additional File 5. Biomass composition of three metabolic models *i*MT1174, *i*JR904, and *i*AF1260.

Table AF5-1 – Biomass composition of three metabolic models *i*MT1174, *i*JR904, and *i*AF1260.

**Metabolites**	**Stoichiometric coefficients (in mmol/g-DW)***		
	**Biomass of *i*MT1174**	**Biomass of *i*JR904**	**Biomass of *i*AF1260**
L-Alanine	-6.59×10^{-1}	-4.88×10^{-1}	-5.14×10^{-1}
L-Arginine	-1.27×10^{-1}	-2.81×10^{-1}	-2.96×10^{-1}
L-Asparagine	-1.42×10^{-1}	-2.29×10^{-1}	-2.41×10^{-1}
L-Aspartate	-1.41×10^{-1}	-2.29×10^{-1}	-2.41×10^{-1}
L-Cysteine	-6.26×10^{-2}	-8.70×10^{-2}	-9.16×10^{-2}
L-Glutamate	-1.38×10^{-1}	-2.50×10^{-1}	-2.63×10^{-1}
L-Glutamine	-1.36×10^{-1}	-2.50×10^{-1}	-2.63×10^{-1}
Glycine	-9.03×10^{-1}	-5.82×10^{-1}	-6.13×10^{-1}
L-Histidine	-4.72×10^{-2}	-9.00×10^{-2}	-9.47×10^{-2}
L-Isoleucine	-1.72×10^{-1}	-2.76×10^{-1}	-2.91×10^{-1}
L-Leucine	-2.67×10^{-1}	-4.28×10^{-1}	-4.51×10^{-1}
L-Lysine	-1.80×10^{-1}	-3.26×10^{-1}	-3.43×10^{-1}
L-Methionine	-7.96×10^{-2}	-1.46×10^{-1}	-1.54×10^{-1}
L-Phenylalanine	-8.57×10^{-2}	-1.76×10^{-1}	-1.76×10^{-1}
L-Proline	-1.52×10^{-1}	-2.10×10^{-1}	-2.21×10^{-1}
L-Serine	-1.65×10^{-1}	-2.05×10^{-1}	-2.16×10^{-1}
L-Threonine	-1.67×10^{-1}	-2.41×10^{-1}	-2.54×10^{-1}
L-Tryptophan	-2.12×10^{-2}	-5.40×10^{-2}	-5.68×10^{-2}
L-Tyrosine	-5.72×10^{-2}	-1.31×10^{-1}	-1.38×10^{-1}
L-Valine	-2.87×10^{-1}	-4.02×10^{-1}	-4.23×10^{-1}
dATP	-7.20×10^{-3}	-2.47×10^{-2}	-2.62×10^{-2}
dCTP	-1.87×10^{-2}	-2.54×10^{-2}	-2.70×10^{-2}
dTTP	-7.20×10^{-3}	-2.47×10^{-2}	-2.62×10^{-2}
dGTP	-1.87×10^{-2}	-2.54×10^{-2}	-2.70×10^{-2}
CTP	-3.09×10^{-2}	-1.26×10^{-1}	-1.34×10^{-1}
GTP	-2.48×10^{-2}	-2.03×10^{-1}	-2.15×10^{-1}
UTP	-1.99×10^{-2}	-1.36×10^{-1}	-1.44×10^{-1}
AMP	0.00	-1.00×10^{-3}	0.00
Phosphatidylethanolamine	-1.44×10^{-1}	-1.94×10^{-3}	-1.39×10^{-1}
Phosphatidylglycerol	-3.30×10^{-2}	-4.64×10^{-4}	0.00
Cardiolipin	-6.90×10^{-3}	-1.29×10^{-4}	0.00
Phosphatidylserine	0.00	-5.20×10^{-5}	0.00

* Negative entries represent consumption of the metabolites (i.e., educts), while positive entries stand for net production of the metabolites (i.e., products).

Table AF5-1 – Biomass composition of three metabolic models *i*MT1174, *i*JR904, and *i*AF1260. (continued)

Metabolites	Stoichiometric coefficients (in mmol/g-DW)*		
	Biomass of *i*MT1174	Biomass of *i*JR904	Biomass of *i*AF1260
NAD+	-3.00×10^{-3}	-2.15×10^{-3}	-1.83×10^{-3}
NADP+	-2.70×10^{-3}	-1.30×10^{-4}	-4.47×10^{-4}
NADH	0.00	-5.00×10^{-5}	0.00
NADPH	0.00	-4.00×10^{-4}	0.00
Succinyl-CoA	0.00	-3.00×10^{-6}	0.00
Acetyl-CoA	0.00	-5.00×10^{-5}	0.00
CoA	-2.60×10^{-3}	-6.00×10^{-6}	-5.76×10^{-4}
2-Octaprenyl-6-hydroxyphenol	0.00	0.00	-2.23×10^{-4}
S-Adenosyl-L-methionine	0.00	0.00	-2.23×10^{-4}
Tetrahydrofolate	-4.50×10^{-3}	0.00	-2.23×10^{-4}
5-Methyltetrahydrofolate	0.00	-5.00×10^{-2}	0.00
10-Formyltetrahydrofolate	0.00	0.00	-2.23×10^{-4}
5,10-Methylenetetrahydrofolate	0.00	0.00	-2.23×10^{-4}
Riboflavin	0.00	0.00	-2.23×10^{-4}
FMN	-4.40×10^{-3}	0.00	0.00
FAD	-2.60×10^{-3}	-1.00×10^{-5}	-2.23×10^{-4}
Menaquinone	-2.30×10^{-3}	0.00	0.00
Ubiquinone-8	-2.80×10^{-3}	0.00	0.00
Phylloquinone	-4.40×10^{-3}	0.00	0.00
Heme A	-2.30×10^{-3}	0.00	0.00
Heme	0.00	0.00	-2.23×10^{-4}
Siroheme	-2.20×10^{-3}	0.00	-2.23×10^{-4}
Ferricytochrome c	-2.30×10^{-3}	0.00	0.00
Ferricytochrome b1	-3.20×10^{-3}	0.00	0.00
Coproporphyrinogen I	-3.00×10^{-3}	0.00	0.00
Thiamin diphosphate	0.00	0.00	-2.23×10^{-4}
NH3	0.00	0.00	-1.18×10^{-2}
Sulfate	0.00	0.00	-3.95×10^{-3}
Cl-	0.00	0.00	-4.74×10^{-3}
Cobalt ion	0.00	0.00	-3.16×10^{-3}
Fe2+	0.00	0.00	-7.11×10^{-3}
UDP-N-acetylmuramate	-1.83×10^{-1}	0.00	0.00
UDP-N-acetyl-D-glucosamine	-2.37×10^{-1}	0.00	0.00
D-Alanyl-D-alanine	-3.46×10^{-1}	0.00	0.00
meso-2,6-Diaminoheptanedioate	-2.08×10^{-1}	0.00	0.00
D-Glutamate	-1.85×10^{-1}	0.00	0.00
UDP-D-galactose	-3.79×10^{-2}	0.00	0.00
UDP-glucose	0.00	-3.00×10^{-3}	0.00
Hexadecanoyl-[acp]	-6.28×10^{-1}	0.00	0.00
Carbohydrate	0.00	-8.40×10^{-3}	0.00

* Negative entries represent consumption of the metabolites (i.e., educts), while positive entries stand for net production of the metabolites (i.e., products).

Table AF5-1 – Biomass composition of three metabolic models *i*MT1174, *i*JR904, and *i*AF1260. (continued)

**Metabolites**	**Stoichiometric coefficients (in mmol/g-DW)***		
	**Biomass of *i*MT1174**	**Biomass of *i*JR904**	**Biomass of *i*AF1260**
Crosslinked peptideglycan	0.00	-5.25×10^{-2}	0.00
di-trans,poly-cis-Undecaprenyl diphosphate	0.00	0.00	-5.50×10^{-5}
ATP	-4.54×10^{1}	-3.02×10^{1}	-3.02×10^{1}
H_2O	-4.54×10^{1}	-3.00×10^{1}	-2.47×10^{1}
ADP	4.54×10^{1}	3.00×10^{1}	3.00×10^{1}
Orthophosphate	4.54×10^{1}	3.00×10^{1}	3.00×10^{1}
Diphosphate	1.45×10^{-1}	7.30×10^{-1}	7.74×10^{-1}
Acyl-carrier protein	6.27×10^{-1}	0.00	0.00
D-Alanine	1.73×10^{-1}	0.00	0.00
UDP	2.75×10^{-1}	0.00	0.00
UMP	1.83×10^{-1}	0.00	0.00
H^+	0.00	3.00×10^{1}	3.00×10^{1}

* Negative entries represent consumption of the metabolites (i.e., educts), while positive entries stand for net production of the metabolites (i.e., products).
