# Supplementary material for: Genome-scale metabolic model of Rhodococcus jostii RHA1 (iMT1174) to study the accumulation of storage compounds during nitrogen-limited condition
Source: BMC Syst Biol. 2015 Aug 7;9:43. doi: 10.1186/s12918-015-0190-y (PMC4528721; doi:10.1186/s12918-015-0190-y)
Supplement: Additional file 6: — Central metabolic pathways of R. jostii RHA1. (PDF 329 kb) [file 12918_2015_190_MOESM6_ESM.pdf]

Central metabolic pathways of *R. jostii* RHA1

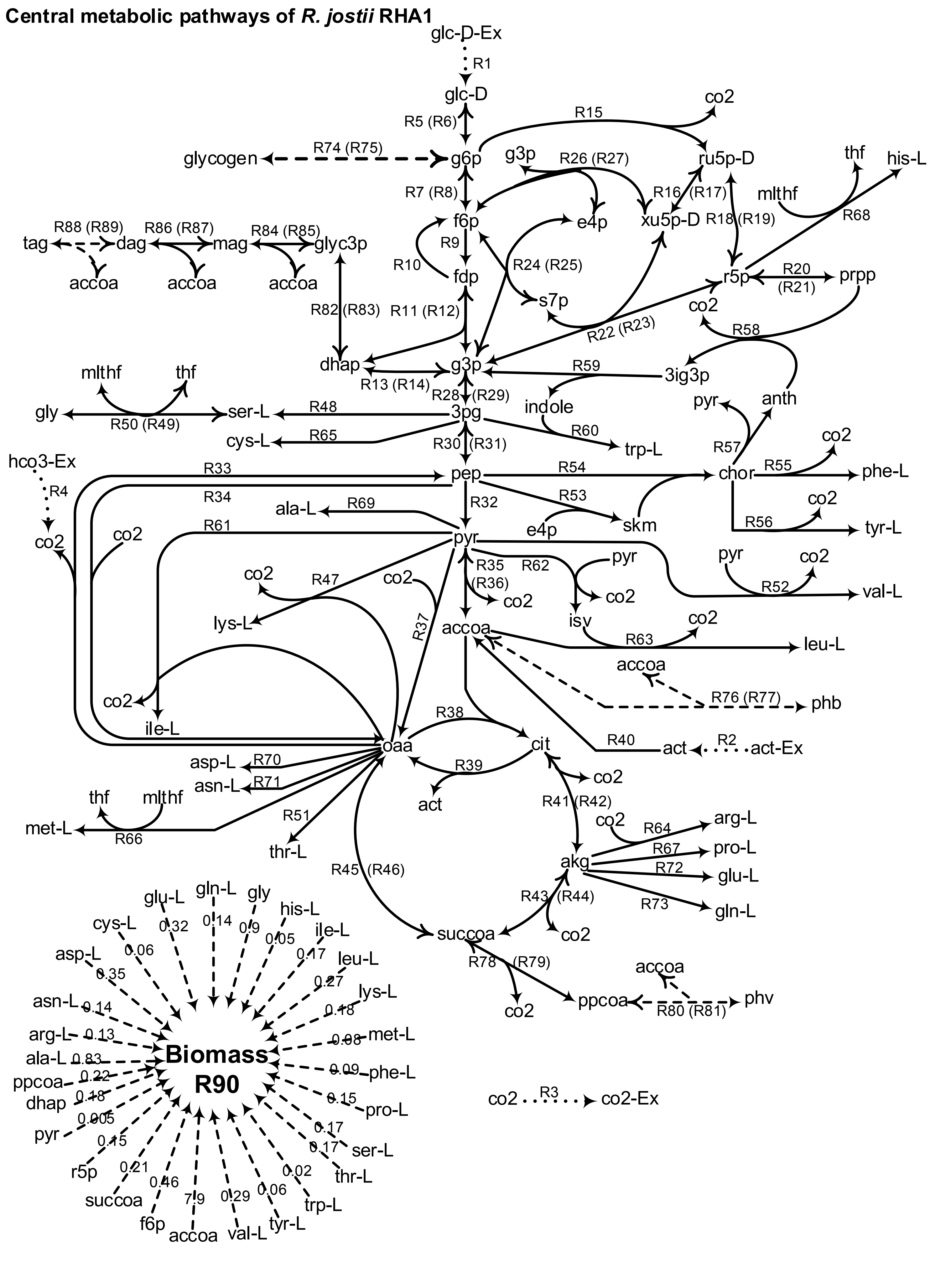

Following is the list of the metabolites involved in the above *R. jostii* RHA1 network:

glc-D-Ex: extracellular alpha-D-Glucose, glc-D: alpha-D-Glucose, act-Ex: extracellular acetate, act: acetate, co2-Ex: extracellular carbon dioxide, co2: carbon dioxide, hco3-Ex: extracellular bicarbonate, g6p: alpha-D-Glucose 6-phosphate, f6p: beta-D-Fructose 6-phosphate, fdp: beta-D-Fructose 1,6-bisphosphate, g3p: D-Glyceraldehyde 3-phosphate, dhap: Glycerone phosphate, ru5p-D: D-Ribulose 5-phosphate, xu5p-D: D-Xylulose 5-phosphate, r5p: D-Ribose 5-phosphate, prpp: 5-Phospho-alpha-D-ribose 1-diphosphate, s7p: Sedoheptulose 7-phosphate, e4p: D-Erythrose 4-phosphate, 3pg: 3-Phospho-D-glycerate, pep: Phosphoenolpyruvate, pyr: Pyruvate, oaa: Oxaloacetate, accoa: Acetyl-CoA, cit: Citrate, akg: 2-Oxoglutarate, succoa: Succinyl-CoA, lys-L: L-Lysine, ser-L: L-Serine, gly: Glycine, mlthf: 5,10-Methylenetetrahydrofolate, thf: Tetrahydrofolate, thr-L: L-Threonine, val-L: L-Valine, skm: Shikimate, chor: Chorismate, phe-L: L-Phenylalanine, tyr-L: L-Tyrosine, anth: Anthranilate, 3ig3p: Indoleglycerol phosphate, indole: Indole, trp-L: L-Tryptophan, ile-L: L-Isoleucine, isv: ISV, leu-L: L-Leucine, arg-L: L-Arginine, cys-L: L-Cysteine, met-L: L-Methionine, pro-L: L-Proline, his-L: L-Histidine, ala-L: L-Alanine, asp-L: L-Aspartate, asn-L: L-Asparagine, glu-L: L-Glutamate, gln-L: L-Glutamine, glycogen: Glycogen, phb: Poly-beta-hydroxybutyrate, ppcoa: Propanoyl-CoA, phv: Poly-beta-Hydroxyvalerate, glyc3p: sn-Glycerol 3-phosphate, mag: sn-Glycerol1, dag: sn-Glycerol2, and tag: Triacylglycerol.

Following is the list of the reactions, along with their KEGG IDs, involved in the above *R. jostii* RHA1 network; b in the KEGG IDs stands for the backward reaction.

R1: A00576, R2: A00680, R3: A00251, R4: A00677, R5: R01786, R6: R01786, R7: R02740, R8: R02740b, R9: R04779, R10: R04780, R11: R01070, R12: R01070b, R13: R01015, R14: R01015b, R15: lumped reaction, R16: R01529, R17: R01529b, R18: R01056, R19: R01056b, R20: R01049, R21: R01049b, R22: R01641b, R23: R01641, R24: R01827, R25: R01827b, R26: R01830b, R27: R01830, R28: R01015, R29: R01015b, R30: lumped reaction, R31: lumped reaction, R32: R00200, R33: R00431, R34: R00345, R35: R01196, R36: R01196b, R37: R00344, R38: R00351, R39: R00362, R40: R00235, R41: lumped reaction, R42: lumped reaction, R43: A00619, R44: A00619b, R45: R00405b, R46: R00405, R47: L-Lysine biosynthesis (lumped reaction), R48: L-Serine biosynthesis (lumped reaction), R49: R00945, R50: R00945b, R51: L-Threonine biosynthesis (lumped reaction), R52: L-Valine biosynthesis (lumped reaction), R53: Shikimate biosynthesis (lumped reaction), R54: Chorismate biosynthesis (lumped reaction), R55: L-Phenylalanine biosynthesis (lumped reaction), R56: L-Tyrosine biosynthesis (lumped reaction), R57: R00985, R58: lumped reaction, R59: R02340, R60: R00674, R61: L-Isoleucine biosynthesis (lumped reaction), R62: L-Leucine biosynthesis (lumped reaction), R63: L-Leucine biosynthesis (lumped reaction), R64: L-Arginine biosynthesis (lumped reaction), R65: L-Cysteine biosynthesis (lumped reaction), R66: L-Methionine biosynthesis (lumped reaction), R67: L-Proline biosynthesis (lumped reaction), R68: L-Histidine biosynthesis (lumped reaction), R69: L-Alanine biosynthesis (lumped reaction), R70: L-Aspartate biosynthesis (lumped reaction), R71: L-Asparagine biosynthesis (lumped reaction), R72: L-Glutamate biosynthesis (lumped reaction), R73: L-Glutamine biosynthesis (lumped reaction), R74: R00959b, R75: R00959, R76: R00238, R77: R00238b, R78: R00833, R79: R00833b, R80: A00040, R81: A00040b, R82: R01011, R83: R01011b, R84: Triacylglycerol biosynthesis (lumped reaction), R85: Triacylglycerol biosynthesis (lumped reaction), R86: Triacylglycerol biosynthesis (lumped reaction), R87: Triacylglycerol biosynthesis (lumped reaction), R88: Triacylglycerol biosynthesis (lumped reaction), R89: Triacylglycerol biosynthesis (lumped reaction), and R90: A00000.
